# Identification of immunogenic HLA class I and II neoantigens using surrogate immunopeptidomes

**DOI:** 10.1126/sciadv.ado6491

**Published:** 2024-09-18

**Authors:** Serina Tokita, Minami Fusagawa, Satoru Matsumoto, Tasuku Mariya, Mina Umemoto, Yoshihiko Hirohashi, Fumitake Hata, Tsuyoshi Saito, Takayuki Kanaseki, Toshihiko Torigoe

**Affiliations:** ^1^ Department of Pathology, Sapporo Medical University, Sapporo, Japan.; ^2^ Joint Research Center for Immunoproteogenomics, Sapporo Medical University, Sapporo, Japan.; ^3^ Department of Surgery, IMS Sapporo Digestive Disease Center General Hospital, Sapporo, Japan.; ^4^ Department of Obstetrics and Gynecology, Sapporo Medical University, Sapporo, Japan.; ^5^ Department of Surgery, Sapporo Dohto Hospital, Sapporo, Japan.

## Abstract

Neoantigens arising from somatic mutations are tumor specific and induce antitumor host T cell responses. However, their sequences are individual specific and need to be identified for each patient for therapeutic applications. Here, we present a proteogenomic approach for neoantigen identification, named Neoantigen Selection using a Surrogate Immunopeptidome (NESSIE). This approach uses an autologous wild-type immunopeptidome as a surrogate for the tumor immunopeptidome and allows human leukocyte antigen (HLA)–agnostic identification of both HLA class I (HLA-I) and HLA class II (HLA-II) neoantigens. We demonstrate the direct identification of highly immunogenic HLA-I and HLA-II neoantigens using NESSIE in patients with colorectal cancer and endometrial cancer. Fresh or frozen tumor samples are not required for analysis, making it applicable to many patients in clinical settings. We also demonstrate tumor prevention by vaccination with selected neoantigens in a preclinical mouse model. This approach may benefit personalized T cell–mediated immunotherapies.

## Teaser

Immunogenic neoantigens can be efficiently identified using surrogate immunopeptidomes without using tumor immunopeptidomes.

## INTRODUCTION

T cells distinguish between cancer and normal cells by recognizing the neoantigens presented by HLA molecules. Neoantigens arising from somatic nonsynonymous mutations in cancer cells can be recognized by circulating T cells, as they are not affected by central T cell tolerance (*1*). Neoantigens play a critical role in T cell activation and have potential clinical applications as targets for T cell–based tumor immunotherapies, such as vaccination (*2*–*12*). Meanwhile, neoantigens are individual specific, and only a small fraction of mutations give rise to immunogenic neoantigens recognized by host T cells (*13*, *14*). Therefore, immunogenic neoantigens must be identified from a large number of mutations in each patient for therapeutic application. Currently, in silico algorithms that predict major histocompatibility complex (MHC) binding are widely used to identify neoantigens, but they are often compromised by a large number of false positives and require further experimental validation with patient material to reveal clinically relevant true neoantigens (*15*, *16*).

Recently, screening methods with improved specificity and sensitivity have been developed. A proteogenomic approach combining affinity purification with sequencing of human leukocyte antigen (HLA)–bound peptides using mass spectrometry (MS) leads to the identification of immunogenic neoantigens directly from human tissue samples (*17*–*23*). On the other hand, tumor-infiltrating lymphocytes (TILs) often recognize neoantigens and can be used as sensitive probes to identify immunogenic neoantigens (*24*, *25*). However, these approaches require fresh or fresh-frozen tumor tissue, or live TILs, which are not always available in clinical settings, limiting the number of patients who can benefit from these analytical options. Furthermore, neoantigens are displayed by both HLA-I and HLA-II molecules, and neoantigen-reactive CD4^+^ T cells are likely to play an indispensable role in T cell–mediated tumor rejection in vivo (*26*–*30*). However, HLA-II heterodimers are highly polymorphic, peptides displayed by HLA-II vary in length, and many tumor cells of epithelial origin lack surface expression of HLA-II, making the prediction of HLA-II neoepitopes a challenge. HLA-II binding, unlike HLA-I binding, is often not considered in the selection of neoantigen sequences in neoantigen vaccine clinical trials (*2*, *4*, *6*–*8*, *10*, *11*). Therefore, the development of a method to identify clinically relevant both HLA-I and II neoantigens from as many patients as possible is an urgent issue for the practical application of personalized medicine targeting neoantigens.

Here, we present a method for neoantigen identification using a unique proteogenomic pipeline that differs from currently available tools. This approach enables the identification of HLA-presented neoantigens by combining surrogate immunopeptidomes obtained from nontumor autologous tissues, such as peripheral blood–derived mononuclear cells (PBMCs), with somatic gene mutation data obtained from tumor samples. This approach allows HLA-agnostic identification of both HLA-I and HLA-II neoantigens and eliminates the need for fresh or fresh-frozen tumor samples, thereby making it applicable to many patients in clinical settings.

## RESULTS

### Development of an approach for neoantigen identification using surrogate immunopeptidomes

**Fig. 1.**
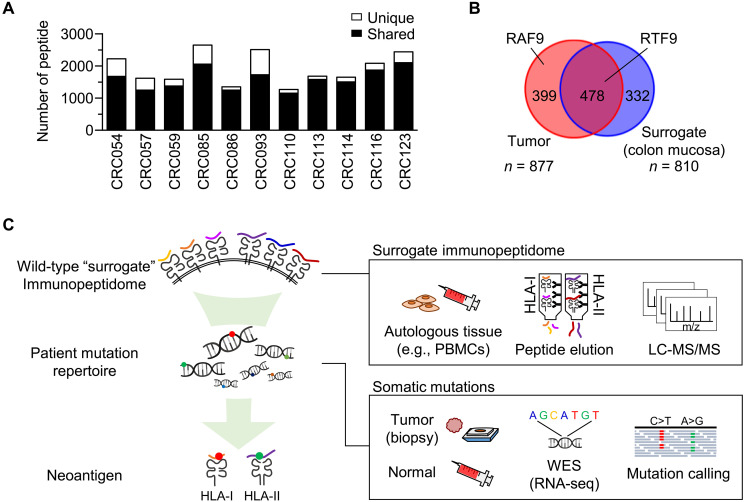
Neoantigen identification using a surrogate immunopeptidome. (**A**) Bar chart showing the overlap of HLA-A24 ligands in samples with the same HLA but from different patients with CRC. HLA-A24 ligands were independently obtained from the tumor tissues of 11 different HLA-A*24:02–positive patients with CRC. Peptides were eluted with an HLA-A24 antibody (C7709A2) and sequenced by MS (false discovery rate 0.01). The number of peptide sequences shared by two or more samples is shown in black, and the number of sequences unique to each sample is shown in white. (**B**) Venn diagram showing the number of overlapping HLA-A24 ligands between the CRC and autologous normal (nontumor) colon mucosa tissues in a patient with CRC (CRC111) based on the data reported by Hirama *et al.* (*22*). A reported neoantigen (RYLAVAAVF, RAF9) was found exclusively in the tumor immunopeptidome, while its wild-type counterpart (RYLTVAAVF, RTF9) was detected in both tumor and normal immunopeptidomes. (**C**) Overview of neoantigen identification by NESSIE.

Conventional proteogenomic approaches using MS comprehensively scan the immunopeptidomes of tumor cells or tissues, revealing thousands of HLA-presented peptide sequences per sample, mostly consisting of nonmutated wild-type peptides. In contrast to neoantigens, wild-type peptides are shared between HLA-matched samples from the same or different individuals. Approximately 69 to 94% of the MS-identified HLA-A24 peptides were shared by two or more samples from 11 patients with colorectal cancer (CRC) (Fig. 1A and table S1). This result is consistent with the notion that HLA peptidomes originate from a limited, but not random, fraction of the protein-coding region of the genome (*31*). In addition, tumor immunopeptidomes often contain both neoantigens and their wild-type counterpart peptides simultaneously (*17*–*19*, *21*–*23*, *32*, *33*). We have previously analyzed a tumor sample from a patient with CRC and reported an immunogenic neoantigen, RAF9 (*22*). We also found its wild-type counterpart (RTF9) in the immunopeptidome of the nontumor colon mucosa of the same patient (Fig. 1B). Therefore, we hypothesized that HLA-matched wild-type peptidomes, rather than tumor peptidomes, could be used as a peptide pool for neoantigen identification in combination with gene mutation data. Here, we present an approach named “Neoantigen Selection using a Surrogate Immunopeptidome (NESSIE)” (Fig. 1C). This approach searches the wild-type immunopeptidome for peptides whose positions match the missense mutations determined by whole-exome sequencing (WES). Tumor tissues are used only to obtain patient-specific mutation data. This approach uses wild-type immunopeptidomes as surrogates for tumor immunopeptidomes and detects candidate neoantigens without using in silico prediction algorithms.

### Identification of immunogenic neoantigens in clinical samples using NESSIE

**Fig. 2.**
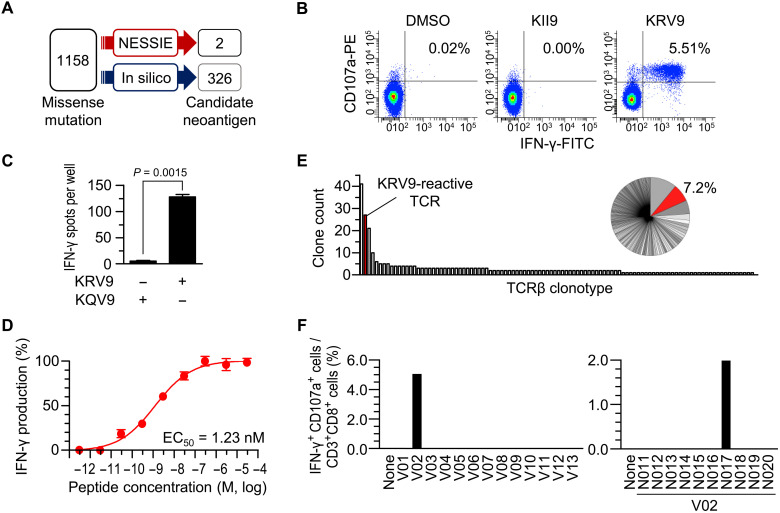
Identification of immunogenic neoantigens using NESSIE. (**A**) Detection of HLA-A*02:01 neoantigens in CRC135 by NESSIE or in silico prediction. NESSIE search was performed using the HLA-A*02:01 peptidome data from autologous colon mucosa with missense mutation data. The mutations with a %rank score of NetMHCpan4.1 < 2.0 with gene TPM > 1 were counted as in silico–predicted candidate neoantigens. (**B**) Flow cytometry of CRC135 bulk CD8^+^ TILs in response to the two candidate neoantigens (KII9 and KRV9) detected by NESSIE. The numbers indicate the frequency of the upper right quadrant in all CD3^+^ CD8^+^ TILs. Data are representative of four independent experiments. DMSO, dimethyl sulfoxide. (**C**) IFN-γ ELISPOT assay of a KRV9-reactive CD8^+^ T cell clone (4H11) in response to a neoantigen (KRV9) or wild-type (KQV9) peptide. Data are shown as the means ± SEM (*n* = 3). *P* value was calculated using a two-tailed paired *t* test. (**D**) Functional avidity of the KRV9-reactive CD8^+^ T cell clone measured by IFN-γ ELISPOT assay with a range of indicated concentrations of KRV9 peptide. The number of positive spots was normalized (%). Data are shown as the means ± SEM (*n* = 3). (**E**) Distribution (bar graph) and frequency (pie chart) of the TCRβ clonotypes of CRC135 bulk TILs (167 clonotypes in total). The TCRβ clonotype of the KRV9-reactive CD8^+^ T cell clone is shown in red. (**F**) Screening of in silico–predicted neoantigens using tandem IVTT. Bar graphs representing the frequency of IFN-γ^+^ CD107a^+^ cells in the CD3^+^ CD8^+^ CRC135 bulk TILs shown in fig. S2 (C and D). TIL responses to the first-round IVTT products of 13 vectors (V01 to V13) encoding all 126 candidate neoantigens detected by in silico prediction with %rank NetMHC scores below 0.5 (left) or the second-round IVTT products (candidate neoantigens, N011 to N020) of vector V02 (right) were measured.

To evaluate the feasibility of NESSIE in detecting clinically relevant neoantigens, we analyzed a patient with CRC (CRC135) carrying 1158 missense mutations. NESSIE search of the wild-type HLA-A*02:01 peptidome of the autologous nontumor colon mucosa with tumor missense mutation data determined by WES revealed two candidate neoantigens (Fig. 2A and table S2). In contrast, an in silico prediction algorithm (NetMHCpan4.1) combined with RNA sequencing (RNA-seq) gene expression data predicted 326 candidate neoantigens potentially presented by HLA-A*02:01 from the same mutation data (see Materials and Methods for details). The following experiment demonstrated that in vitro–expanded bulk CD8^+^ TILs responded to one of the two candidate neoantigens detected by NESSIE (Fig. 2B). We further established CD8^+^ T cell clones recognizing the KRV9 neoantigen. The TIL-derived clones specifically recognized the KRV9 neoantigen, but not its wild-type counterpart (KQV9), demonstrating their neoantigen specificity to discriminate the single–amino acid substitution (STT3A, p.Q294R) (Fig. 2C and fig. S1). The T cell receptor (TCR) of the clone 4H11 showed high functional avidity with median effective concentration (EC_50_) of 1.23 nM for the recognition of KRV9 (Fig. 2D). We also evaluated the frequency of the neoantigen-reactive T cells in vivo using the TCR sequencing data. TCR clonotype analysis showed that the frequency of T cells with the KRV9-reactive TCR accounted for approximately 7.2% of the bulk TILs, implying their expansion in the tumor microenvironment (TME; Fig. 2E).

For comparison, we also asked how many of the in silico–predicted neoantigens could be recognized by the bulk TILs. To screen a large number of candidate neoantigens, we used a “tandem in vitro transcription and translation (IVTT)” method (fig. S2). Here, a DNA vector contained up to 10 neoantigen units, each unit consisting of a neoantigen sequence with an upstream T7 promoter, ribosome binding site, start codon and FLAG sequence, and a downstream stop codon and 3′ untranslated region (3′UTR) stem loop. Therefore, either a mixed pool of neoantigen peptides (for first-round screening) or a single neoantigen peptide of interest (for second-round screening) can be produced from the vector by IVTT. The in silico–predicted neoantigens consisted of 126 strong binders (%rank NetMHC scores below 0.5) and 200 weak binders (%rank NetMHC scores below 2.0), and we evaluated all 126 strong binders using the tandem IVTT method. The IVTT products were coincubated with T2 cells and evaluated by TIL recognition. As a result, we found that a single vector (V02) was recognized among the 13 vectors in the first round of screening, and the N017 peptide was found as a single immunogenic neoantigen in the second round of screening (Fig. 2F). The N017 peptide was KRV9 (table S2). Although we cannot exclude the possibility that some neoantigens may have been missed because of the skewed TCR repertoire in in vitro expanded TILs or because IVTT efficiency may vary depending on peptide sequences, KRV9 was the only immunogenic neoantigen detected by in silico prediction in our screen. Thus, both NESSIE and in silico prediction identified the highly immunogenic neoantigen. NESSIE detected it as one of the 2 candidates, whereas in silico prediction detected it as one of the 126 candidates.

### Neoantigen identification using patient peripheral blood

**Fig. 3.**
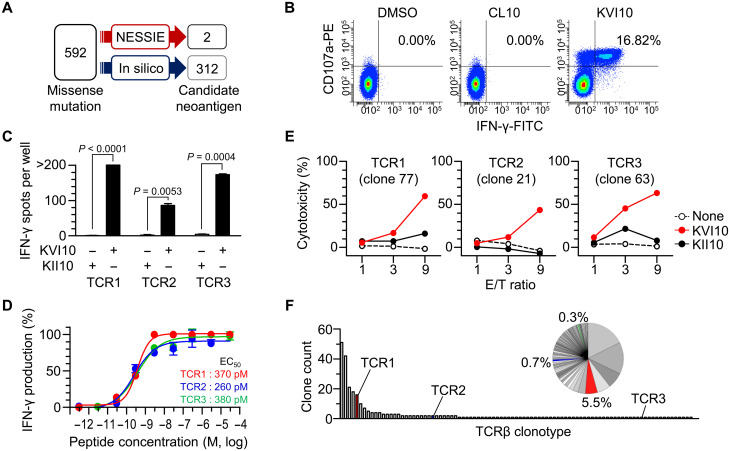
Neoantigen identification using NESSIE with peripheral blood. (**A**) Detection of HLA-I neoantigens in UTE003 by NESSIE or in silico prediction. NESSIE search was performed using the pan HLA-I peptidome data from autologous LCL with missense mutation data. The mutations with a %rank score of NetMHCpan4.1 < 2.0 with gene TPM > 1 were counted as in silico–predicted candidate neoantigens. (**B**) Flow cytometry of UTE003 bulk CD8^+^ TILs in response to the two candidate neoantigens (CL10 and KVI10) detected by NESSIE. The numbers indicate the frequency of the upper right quadrant in all CD3^+^ CD8^+^ TILs. Data are representative of five independent experiments. (**C**) IFN-γ ELISPOT assay of KVI10-reactive CD8^+^ T cell clones with different TCRαβ clonotypes (TCR1, clone 77; TCR2, clone 21; TCR3, clone 63) in response to a neoantigen (KVI10) or wild type (KII10) peptide. Data are shown as the means ± SEM (*n* = 3). *P* values were calculated using a two-tailed paired *t* test. (**D**) Functional avidities of the CD8^+^ T cell clones measured by IFN-γ ELISPOT assay with a range of indicated concentrations of KVI10 peptide. The number of positive spots was normalized (%). Data are shown as the means ± SEM (*n* = 3). (**E**) LDH-release cytotoxicity of the indicated clones with different TCR clonotypes cultured with T2 cells pulsed with 1 μM of the indicated peptide. *X* axis, effector/target (E/T) ratio; *y* axis, LDH release (%) by target cells. Data are representative of two independent experiments. (**F**) Distribution (bar graph) and frequencies (pie chart) of the TCRβ clonotypes of UTE003 bulk TILs (93 clonotypes in total). TCRβ clonotypes of the KVI10-reactive CD8^+^ T cell clones (TCR1, TCR2, and TCR3) are indicated in red, blue, and green, respectively.

Normal tissue, especially peripheral blood, is relatively easy to obtain with minimal invasion, making it an accessible sample for analysis. Therefore, we analyzed a patient with endometrioid cancer (UTE003) and searched for neoantigens using the pan HLA-I peptidome of a patient PBMC-derived lymphoblastoid cell line (LCL) with tumor missense mutation data. The use of patient LCL as a surrogate immunopeptidome revealed two candidate neoantigens from 592 missense mutations (Fig. 3A). Bulk CD8^+^ TILs readily responded to one of the two candidates, which likely presented by HLA-A*02:01 (Fig. 3B and table S3). Sixteen TIL-derived CD8^+^ T cell clones with three different TCR clonotypes were subsequently established. They showed neoantigen (KVI10) specificity in discriminating the single–amino acid substitution (Fig. 3C and fig. S3) and similarly high TCR functional avidities (EC_50_: 260 to 380 pM; Fig. 3D). The representative three T cell clones with different TCRs were functionally active, specifically killing the target cells presenting KVI10 (Fig. 3E). Last, TCR clonotype analysis demonstrated the expansion of KVI10-reactive T cells in the TME (Fig. 3F). Thus, KVI10, one of the two neoantigens detected by NESSIE, was highly immunogenic and induced host CD8^+^ T cell responses against tumor cells. These results suggest the potential of peripheral blood as a surrogate immunopeptidome source for efficient neoantigen identification.

### Comparison of NESSIE and conventional prediction methods

**Fig. 4.**
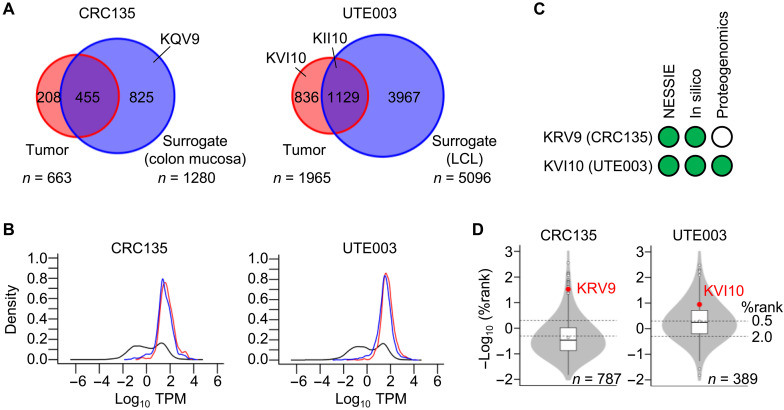
Comparison between NESSIE, in silico prediction, and conventional proteogenomics. (**A**) Venn diagram showing the number of overlapping HLA-A*02:01 ligands between CRC135 tumor and autologous normal colon mucosa (left), and the number of overlapping HLA-I ligands between the UTE003 tumor and autologous LCLs (right). In CRC135, wild-type peptide (KLNPQQFEV, KQV9) of the immunogenic neoantigen (KLNPQRFEV, KRV9) was found exclusively in the surrogate immunopeptidome. In UTE003, wild-type peptide (KLWDIINVNI, KII10) of the immunogenic neoantigen (KLWDIVNVNI, KVI10) was detected in both tumor and the surrogate immunopeptidomes. (**B**) Kernel density estimation of CRC135-tumor transcripts (left) and UTE003-tumor transcripts (right) showing similarity in source gene distribution between the tumor and surrogate immunopeptidomes. Black line indicates the total transcriptome. Red and blue lines indicate the distribution of the source genes of the tumor and surrogate immunopeptidomes, respectively. (**C**) Summary of identified neoantigens. In silico, in silico prediction as described in Figs. 2A and 3A; Proteogenomics, a conventional proteogenomic approach searching the tumor immunopeptidomes for neoantigens using MS; green circle, detected; empty circle, not detected. (**D**) Violin plot showing the %rank scores for the immunogenic neoantigens (KRV9 and KVI10) determined by in silico prediction (source gene TPM > 1). In CRC135, KRV9 was ranked 17th among the 326 HLA-A*02:01 candidate neoantigens. In UTE003, KVI10 was ranked 69th among the 312 HLA-I candidate neoantigens. Dashed lines indicate the thresholds for strong (0.5) and weak (2.0) binders, as defined by NetMHCpan4.1.

Next, we asked about the mechanisms of action of NESSIE in the detection of immunogenic neoantigens. Because the identification of neoantigens by NESSIE relies on the presence of wild-type counterparts in the surrogate immunopeptidome, we investigated the properties of the peptides detected in the tumor and surrogate immunopeptidomes. The identified HLA-A*02:01 peptidomes of CRC135 tumor and nontumor colon mucosa tissues contained a total of 663 and 1280 unique peptides, respectively. Similarly, the pan HLA-I peptidomes of UTE003 tumor tissue and LCL contained a total of 1965 and 5096 unique peptides, respectively (Fig. 4A). The cell surface HLA expression of LCL was higher because of the nature of hematopoietic cells, likely resulting in the identification of more than twice as many peptides as in the other samples. Approximately 68.6% (455 of 663) and 57.5% (1129 of 1965) of the CRC135 and UTE003 tumor immunopeptidomes, respectively, were shared with their surrogate immunopeptidomes. Kernel density estimates of CRC135 and UTE003 tumor transcripts for the identified peptides were equally biased toward genes with high expression levels, suggesting similarity in the source gene distribution and sharing of relatively abundant peptides between tumor and surrogate immunopeptidomes (Fig. 4B). We also compared the quality of the UTE003 tumor and LCL immunopeptidomes, as these peptide repertoires may differ because of the difference between the constitutive and immunoproteasomes. However, there were no clear differences, at least with respect to the length distribution of the peptides, the amino acid composition of the C-terminal amino acids, or the proportion of HLA types presenting peptides (fig. S4).

Of the two immunogenic neoantigens identified by NESSIE, KVI10 was detected in the UTE003 tumor immunopeptidome, but KRV9 was not detected in the CRC135 tumor immunopeptidome, meaning that KRV9 could not be detected by conventional proteogenomic analysis of the tumor sample (Fig. 4, A and C). Its wild-type counterpart (KQV9) was exclusively present in the surrogate immunopeptidome, leading to the identification of the KRV9 neoantigen. Meanwhile, in silico prediction using NetMHCpan and RNA-seq data predicted 326 HLA-A*02:01 and 312 HLA-I candidate neoantigens (%rank NetMHC scores below 2.0) in CRC135 and UTE003, respectively (Figs. 2A and 3A). Retrospectively, we found that both KRV9 and KVI10 were predicted as candidate neoantigens with %rank scores below 0.5 (Fig. 4D). This result potentially supports the high sensitivity of in silico prediction for the identification of immunogenic neoantigens, although the specificity may not be high enough (Fig. 2F).

### Identification of immunogenic HLA-II neoantigens

**Fig. 5.**
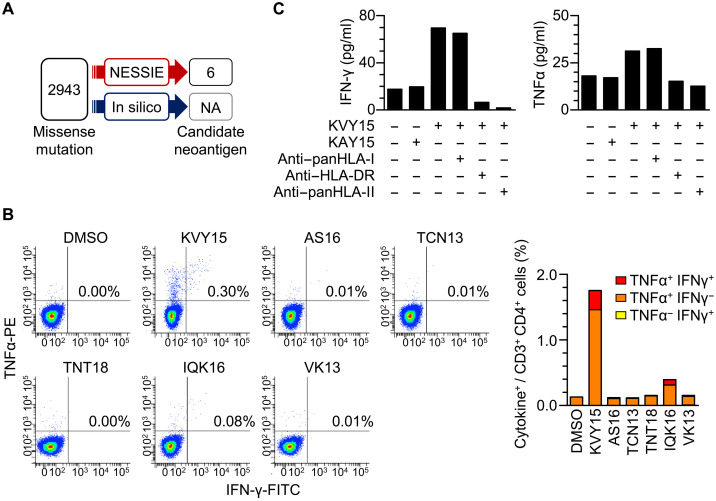
Identification of HLA-II neoantigens using NESSIE. (**A**) Detection of HLA-II neoantigens in CRC066 by NESSIE. NESSIE search was performed using the HLA-II peptidome data from autologous LCL with missense mutation data. NA, untested. (**B**) Flow cytometry of patient CD3^+^ CD4^+^ PBMCs in response to the six candidate neoantigens (KVY15, AS16, TCN13, TNT18, IQK16, and VK13) detected by NESSIE. The numbers indicate the frequency of the upper right quadrant in all CD3^+^ CD4^+^ PBMCs. (**C**) IFN-γ or TNFα ELISA of patient PBMCs in response to a neoantigen (KVY15) or wild-type (KAY15) peptide in the presence of the indicated HLA antibodies.

Recent reports underscore the multiple functions and diverse roles of CD4^+^ T cells in promoting tumor rejection in vivo (*30*, *34*). Unlike tumor cells of epithelial origin, which often lack surface HLA-II expression, LCL constantly expresses HLA-II. Because their HLA types are fully matched to those of the tumor cells, we hypothesized that NESSIE using autologous LCL facilitates the identification of HLA-II neoantigens. To verify this hypothesis, we analyzed a patient with CRC (CRC066) carrying 2943 missense mutations, where tumor cells lacked HLA-II expression (fig. S5). The surrogate HLA-II peptidome from the patient-derived LCL contained 3269 unique sequences whose repertoire showed a pattern of length distribution and source protein cellular localization different from those of the HLA-I peptidomes. NESSIE search of the autologous HLA-II peptidome ultimately detected six candidate neoantigens (Fig. 5A and table S4). In silico prediction was not performed because of the aforementioned issues of HLA-II antigen identification caused by highly polymorphic HLA-II heterodimers and promiscuous peptides in length.

Here, we evaluated interferon-γ (IFN-γ) production by the patient’s PBMCs. We found that patient PBMCs contained a CD4^+^ T cell fraction that responded to two of the six candidate neoantigens (Fig. 5B). After stimulation with a mixture of the six candidate neoantigens, the patient’s PBMCs produced IFN-γ and tumor necrosis factor–α (TNFα) in response to KVY15 and, to a lesser extent, IQK16 peptides. IFN-γ production in response to KVY15 was decreased in the presence of pan–HLA-II or HLA-DR antibodies, suggesting HLA-DR presentation of the KVY15 peptide (Fig. 5C). Therefore, we considered that the candidate neoantigens detected by NESSIE contained immunogenic HLA-II neoantigens that induced patient CD4^+^ T cells. The PBMC response to the neoantigens after 2 weeks of in vitro stimulation may indicate the presence of KVY15- and IQK16-reactive memory CD4^+^ T cells in the patient’s circulation. Thus, NESSIE successfully identified clinically relevant HLA-II neoantigens using the surrogate immunopeptidome of autologous LCL within the first round of screening in this patient with CRC.

### NESSIE-identified neoantigens as cancer vaccine

**Fig. 6.**
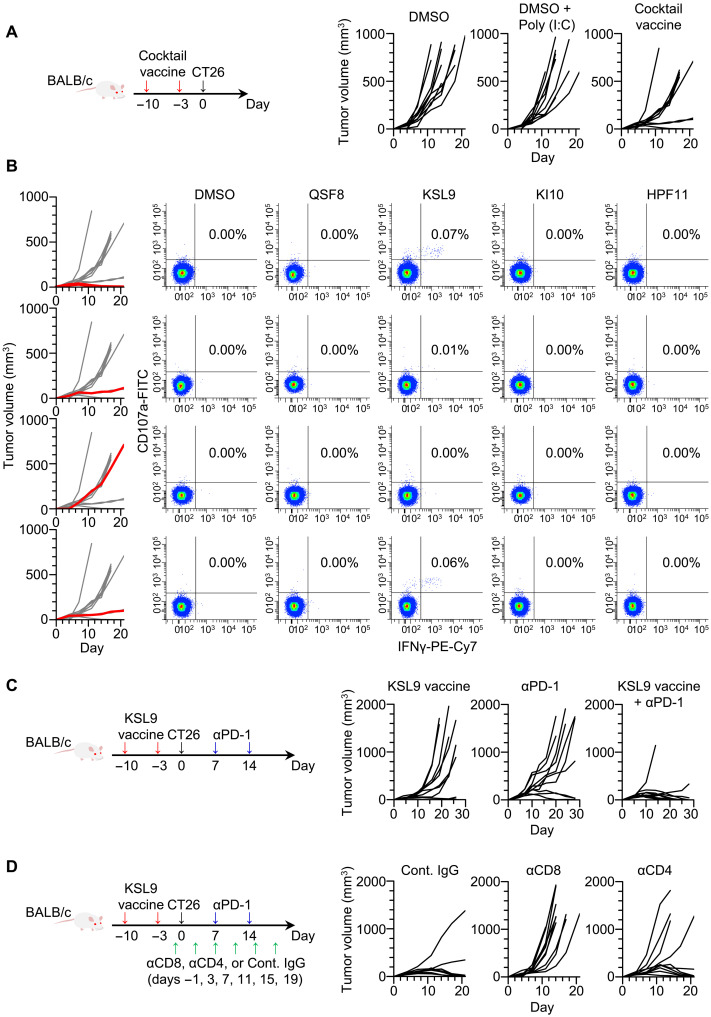
Tumor rejection in vivo by NESSIE-based neoantigen vaccination. (**A**) Syngeneic BALB/c mice were subcutaneously vaccinated with a peptide mixture of four neoantigens detected by NESSIE and subsequently challenged with 5 × 10^6^ CT26. Each line represents the tumor size for each individual (*n* = 10 in all groups). (**B**) Left: CT26 tumor growth curve shown in (A) and spleen cells of the individuals shown in red were analyzed. Right: Flow cytometry of CD8^+^ spleen cells showing intracellular IFN-γ production and CD107a surface expression in response to the indicated neoantigens. The numbers indicate the frequency of the upper right quadrant in all CD3^+^ CD8^+^ spleen cells. (**C**) Tumor rejection by the combined neoantigen vaccine and programmed cell death 1 (PD-1) antibody. Mice were subcutaneously vaccinated with a neoantigen peptide (KSL9) and subsequently challenged with 5 × 10^6^ CT26, followed by the intraperitoneal injection of the PD-1 antibody. Each line represents the tumor size for each individual (*n* = 10 in all groups). (**D**) CD8^+^ T cell–mediated tumor rejection by the combined neoantigen vaccine and PD-1 antibody. Syngeneic BALB/c mice were vaccinated subcutaneously with KSL9 peptide, intraperitoneally with PD-1 antibody and anti-CD8 or anti-CD4 or isotype-matched immunoglobulin G (IgG) antibodies. Mice were injected subcutaneously with 5.0 × 10^6^ CT26 cells on day 0, and their sizes were monitored. Each line represents the tumor size for each individual (*n* = 10 in all groups).

Personalized cancer vaccine with neoantigens, with or without immune checkpoint blockade (ICB), may provide meaningful benefit in a variety of epithelial and nonepithelial tumors (*2*–*12*). Because most current clinical trials vaccinate a mixture of up to 20 to 60 neoantigens, there is a need to develop an approach with both specificity and sensitivity that can be applied to as many patients as possible (*35*). Therefore, we evaluated the efficacy of NESSIE-based cancer vaccines in vivo. In a mouse colon cancer line (CT26), NESSIE using the surrogate immunopeptidome from spleen cells detected four candidate neoantigens that were potentially presented by H-2D^d^, K^d^, or L^d^ (fig. S6 and table S5). Synthetic peptides were prepared for all four candidate neoantigens without further selection. Vaccination with the mixture of the four peptides prevented tumor growth in 3 of 10 syngeneic mice inoculated with the CT26 tumor (Fig. 6A). Analysis of spleen cells demonstrated the induction of CD8^+^ T cell responses against KSL9, one of the four candidate neoantigens, in mice that prevented tumor growth, suggesting tumor rejection mediated by KSL9-reactive T cells (Fig. 6B).

Vaccination with KSL9 alone exhibited T cell–mediated antitumor effects, and moreover, the antitumor effect was further improved when combined with ICB, preventing tumor growth in 9 of 10 treated mice (Fig. 6C). The frequency of KSL9-reactive CD8^+^ T cells in the spleen was increased by the combination, supporting the synergy between vaccination and ICB (fig. S7) (*36*). The antitumor effect of the combination was completely or partially attenuated by the presence of anti-CD8 or anti-CD4 antibodies (Fig. 6D). Together, these data support the advantage of NESSIE in the selection of neoantigens for vaccination. Vaccination induced a neoantigen-reactive T cell response and prevented tumor growth in synergy with ICB, resulting in effective tumor control in vivo.

## DISCUSSION

We present an approach for neoantigen identification that exploits the overlap between tumor and wild-type surrogate immunopeptidomes. A key advantage of NESSIE is the HLA-agnostic identification of neoantigens using autologous surrogate immunopeptidomes. In this study, NESSIE successfully identified three immunogenic neoantigens (KRV9, KVI10, and KVY15) presented by HLA-I or HLA-II in patients with CRC or endometrial cancer. In addition to CD8^+^ T cell responses to HLA-I neoantigens, which mediate tumor rejection by direct killing, induction of CD4^+^ T cell responses to HLA-II neoantigens have been repeatedly reported in preceding neoantigen vaccine clinical trials (*2*–*7*, *10*, *11*). The identification of HLA-II neoantigens enables HLA-II neoantigen vaccination and may benefit patients, as CD4^+^ T cells reactive to the HLA-II neoantigen (KVY15) identified by NESSIE produced IFN-γ and TNFα, suggesting their role in preventing tumor growth.

Our analyses also imply the specificity of NESSIE in identifying immunogenic neoantigens that induce T cell responses in patients. This approach directly identified the KRV9 and KVI10 HLA-I neoantigens that induced the T cells with the second and fifth most abundant TCR clonotypes in the TME of CRC135 and UTE003 tumors, respectively (Figs. 2 and Fig. 3). To our best knowledge, efficient methods identifying those clinically relevant neoantigens eliciting clonally expanded TIL responses have not been established to date. Notably, KRV9 and KVI10 were one of two candidates detected in the first round of screening for each tumor. The potentially high specificity of this approach may be attributed to the identification of peptide sequences that are naturally presented by HLA molecules. Because most of the peptide fragments produced in the cytosol are likely to be degraded by the antigen processing pathway before surface presentation (*37*), precise discrimination of the sequences of naturally presented peptides may increase specificity compared to genome- or transcript-level prediction. In addition, for clinical application as a neoantigen vaccine, the use of NESSIE may reduce the number of false-positive vaccine candidates (those not presented in tumor HLAs in vivo), which may lead to advantages in terms of manufacturing process and cost (e.g., manufacturing of Good Manufacturing Practice grade peptides).

In contrast, this approach should not be able to detect neoantigens that are not accompanied by HLA presentation of their wild-type counterparts. For example, immunogenic neoantigens resulting from frameshift mutations would be undetectable because their wild-type counterparts are virtually absent. This may be true in the case of neoantigens originating from a single–amino acid substitution. Some neoantigens with increased HLA binding affinity after amino acid substitutions are reported to be immunogenic (*38*–*40*). Theoretically, these neoantigen types can be further divided into two subtypes: those accompanied with wild-type HLA presentation, even if it has low HLA binding affinity, and those with none at all. The former should be detected by NESSIE. The latter, however, would not be detected if their wild types were present at all in the HLA immunopeptidome of any tissue. Similarly, if a somatic mutation occurs in an antigen that is expressed exclusively in a particular organ or tissue type, the resulting neoantigen may be undetectable using a surrogate immunopeptidome of different tissue origin. Here, we performed a simple estimation using a published set of immunogenic neoantigens detected in 38 patients and confirmed to induce CD8^+^ T cell responses (*14*). The number of frameshift neoantigens and potential de novo neoantigens was 0% (0 of 56) and 17.9% (10 of 56), respectively. Potential de novo neoantigens were defined as those with wild-type and neoantigen NetMHCpan4.1 scores greater than and less than 2.0, respectively. This category included six of those with a single–amino acid substitution at the HLA-binding anchor residue. However, we believe that not all of these are necessarily undetectable by NESSIE for the reasons discussed above, because MS often detects peptides with poor NetMHC scores. Nevertheless, a maximum of approximately 18% of the immunogenic neoantigens in this set may be missed by NESSIE. Hence, NESSIE may not be an approach that fully covers every type of neoantigens. Although the actual frequency and priority of these types of neoantigens in humans remain to be determined, this limitation should be considered for use in clinical settings.

In summary, the availability of this approach makes it applicable to a wide range of patients with cancer and may readily identify neoantigens that might otherwise be missed by current approaches (Fig. 4). Together with its HLA-agnostic nature, which allows the identification of HLA-II neoantigens, NESSIE may benefit personalized immunotherapy targeting neoantigens. Type of detectable neoantigens, precise specificity, and sensitivity in identifying immunogenic neoantigens should be further validated using a large clinical dataset in a variety of cancer types.

## MATERIALS AND METHODS

### Patient or healthy donor material

This study was approved by the Institutional Review Board (352-1082) and Research Ethics Committee of Sapporo Medical University (29-2-69). Signed informed consent was obtained from all patients and healthy donors. Tumor tissues or adjacent nontumor tissues were sampled during surgery and used to isolate TILs, as described below, or immediately frozen until use. PBMCs were isolated from peripheral blood using Lymphoprep (Cosmo Bio), according to the manufacturer’s instructions.

### Cell preparation and culture

TILs, PBMCs, or cell lines were cultured in complete AIM-V (Gibco) or RPMI (Nacalai Tesque) medium supplemented with 1% penicillin-streptomycin, 1% GlutaMAX (Gibco), 10 mM Hepes, 1 mM sodium pyruvate, 55 μM 2-mercaptoethanol, and 10% human AB serum (Biowest) for AIM-V or 10% fetal bovine serum (Nichirei Biosciences Inc.) for RPMI. For generation of patient LCLs, 5 × 10^6^ PBMCs were cultured in the B95-8 virus–containing supernatant for 2 hours at 37°C. The cells were initially cultured in the complete RPMI medium with cyclosporine A (500 ng/ml), without virus removal. Concentrations of cyclosporine A and virus decreased gradually with the addition of the medium. The cells were cultured for weeks and frozen until further use. CT26 [CRL-2638, American Type Culture Collection (ATCC)] and T2 (CRL-1992, ATCC) cell lines were purchased.

### Isolation of peptides bound to MHC-I and MHC-II

Surgically removed tissues or 5 × 10^8^ immortalized LCLs were used to isolate the MHC-bound peptides, as previously described (*22*). The samples were ground under cryogenic conditions and lysed with the lysate buffer containing 0.25% sodium deoxycholate, 0.2 mM iodoacetamide, 1 mM EDTA, protease inhibitor cocktail (Sigma-Aldrich), 1 mM phenylmethylsulfonyl fluoride, and 1% octyl-β-d glucopyranoside (Dojindo) in Dulbecco's Phosphate-Buffered Saline. The peptides bound to MHC-I or MHC-II were captured as peptide-MHC complexes using affinity chromatography of W6/32 monoclonal antibody (mAb) for pan HLA-I (HB-95, ATCC), IVA12 mAb for pan HLA-II (HB-145, ATCC), BB7.2 mAb for HLA-A2 (HB-82, ATCC), SF1-1.1.10 mAb for H-2K^d^ (HB-159, ATCC), 34-5-8S mAb for H-2D^d^ (HB-102, ATCC), or 28-14-8S mAb for H-2L^d^ (HB-27, ATCC) coupled to CNBr-activated Sepharose 4B (GE Healthcare) overnight. The peptides were then eluted with 0.2% trifluoroacetic acid (TFA) and desalted using Sep-Pak tC18 (Waters) with 28% acetonitrile (ACN) (for MHC-I peptides) or 32% ACN (for MHC-II peptides) in 0.1% TFA and ZipTip U-C18 (Millipore) with 50% ACN in 1% formic acid (FA). The elute was dried by vacuum centrifugation and resuspended in 5% ACN in 0.1% TFA for liquid chromatography–tandem MS (LC-MS/MS) analysis. Hybridomas producing each mAb were cultured in a hybridoma serum-free medium (Gibco) supplemented with 1% penicillin-streptomycin in CELLine Bioreactor Flasks (CL1000, Corning). Condensed mAbs were collected and purified using HiTrap Protein G HP (GE Healthcare).

### LC-MS/MS analysis

Samples containing isolated peptides were loaded into a nanoflow LC (Easy-nLC 1000 system, Thermo Fisher Scientific) coupled online to an Orbitrap mass spectrometer equipped with a nanospray ion source (Q Exactive Plus, Thermo Fisher Scientific). Nanoflow LC separation was performed with a linear gradient of 3 to 30% buffer B (100% ACN and 0.1% FA) with a flow rate of 300 nl/min for 80 min and a 75 μm by 20 cm capillary column with a particle size of 3 μm (NTCC-360, Nikkyo Technos). For MS analysis, survey scan spectra were acquired at a resolution of 70,000 at 200 mass/charge ratio (*m/z*) with an automatic gain control (AGC) target value of 3 × 10^6^ ions and a maximum injection time (IT) of 100 ms, ranging from 350 to 2000 *m/z* (for MHC-I peptides) or 500 to 3000 *m/z* (for MHC-II peptides), with charge states between 1^+^ and 4^+^. A data-dependent top 10 method was used. The MS/MS resolution was 17,500 at 200 *m/z*, with an AGC target value of 1 × 10^5^ ions and a maximum IT of 120 ms. MS/MS data were searched against the reference database using Sequest HT and the Percolator algorithm on the Proteome Discoverer 2.3 platform (Thermo Fisher Scientific). The database contains the GENCODE protein-coding transcript translation sequences (Release 31 or M23). The tolerances of the precursor and fragment ions were set to 10 ppm and 0.02 Da, respectively. Methionine oxidation (+15.995 Da) was selected as the dynamic modification. No specific enzymes were selected for the search. The concatenated target-decoy selection was validated on the basis of *q* values and a false discovery rate of 0.01, which was used in the Percolator node as the peptide detection threshold. Only the 8- to 12-mer and 10- to 25-mer sequences were counted as MHC-I and MHC-II peptides, respectively.

### Whole-exome sequencing

WES and subsequent analysis were performed as previously described (*22*). DNA was extracted using the Allprep DNA/RNA/Protein Kit (Qiagen). Library preparation, sequencing, and mutation calling were performed by Macrogen (Japan). Exome capture libraries were prepared using SureSelect Human All Exon V6 or SureSelect Mouse (Agilent). Sequencing was performed using NovaSeq 6000 (Illumina) with 150-bp paired-end reads and a target depth of 150× coverage per sample. Tumor DNAs and DNAs derived from autologous nontumor colon mucosa (CRC066 and CRC135), PBMCs (UTE003), and spleen cells (CT26), were used for mutation calling. Briefly, the reads were mapped to the reference genome (hg38 or mm10) using BWA (v0.7.10 or v0.7.17), polymerase chain reaction (PCR) duplicates were removed using Picard tools (1.118 or 2.18.2), and exome-based variants were called using GATK (v4.0.5.1). The detected variants were annotated using SnpEff (v4.1 or v4.3t) and filtered using dbSNPs and single-nucleotide polymorphisms from the 1000 Genomes Project. Single-nucleotide variants or indel mutations were called using the Mutect2 tool with default parameter settings. Patient HLA genotypes were determined using the polysolver (*41*).

### RNA-seq

RNA-seq and TCR clonotype analysis were performed as previously described (*22*). Total RNA was isolated from tumor tissues and CT26 using the Allprep DNA/RNA/Protein Kit (Qiagen) or TRIzol Reagent (Invitrogen) with a validated quality of RNA integrity number > 7. Library preparation and sequencing were performed by Macrogen (Japan). Poly A–selected libraries were prepared using the TruSeq Stranded mRNA LT Sample Prep Kit (Illumina). The libraries were sequenced on NovaSeq 6000 (Illumina) with 200 M (tumor) or 40 M (CT26) 100-bp paired-end reads per sample. Read quality was validated, and low-quality reads, adaptor sequences, contaminant DNA, and PCR duplicates were removed using FastQC (v0.11.7) and Trimmomatic (v0.38). The reads were mapped to the human reference genome (hg38 or mm10) using HISAT2 (v2.1.0) and Bowtie2 (v2.3.4.1). The abundance of genes or transcripts was calculated as transcripts per million (TPM) using StringTie (v1.3.4d or v2.1.3b). The resulting data were analyzed using MiXCR to calculate the frequency of TCR clonotypes (*42*).

### Identification of neoantigens by NESSIE

NESSIE requires three multi-FASTA files to search for neoantigens (FASTA-si, FASTA-mt, and FASTA-wt) per patient. FASTA-si contains the peptide sequences of the surrogate immunopeptidome. In this study, MHC-I or MHC-II peptidomes of autologous blood-derived LCLs (UTE003 and CRC066), autologous colon mucosa (CRC135), or BALB/c spleen cells (CT26) served as surrogate immunopeptidomes. FASTA-mt contains full-length sequences of mutant proteins carrying patient-specific amino acid substitutions estimated by WES mutation calling. FASTA-wt contains the wild-type version of the protein sequences listed in the FASTA-mt.

FASTA-si was searched for peptides whose complete sequences were found in FASTA-wt but not in FASTA-mt. Peptides that met these criteria were considered the wild-type counterparts of patient-specific neoantigens. Therefore, their sequences were substituted with the corresponding amino acid sequences registered in FASTA-mt and ultimately counted as candidate neoantigens. Overlapping peptides that had the same amino acid substitution but differed in length according to the N- or C-terminal flanking residues were counted as single peptides. In all cases, peptides with mutations whose gene expression (TPM) determined by tumor RNA-seq was ≤1 were excluded from the candidate neoantigens.

### In silico prediction of neoantigens

Neoantigens were predicted in silico using NetMHCpan4.1 (https://services.healthtech.dtu.dk/services/NetMHCpan-4.1/), along with WES and RNA-seq data. Briefly, %rank scores were calculated using NetMHCpan4.1 for each mutated amino acid determined by WES (*43*). For example, when scoring HLA-A*02:01 neoantigens, scores were calculated for all 8- to 12-mer sequences encompassing the substituted amino acid, and the highest score was used as the %rank score for the mutation. When scoring HLA-I neoantigens without specifying the HLA type, the above scores were calculated for each HLA-I type carried by the patient, and the highest score was used as the %rank score for that mutation. Mutations with a %rank score < 2.0 and with gene expression in the tumor tissue (TPM > 1) were considered as candidate neoantigens.

### TILs and neoantigen-reactive CD8^+^ T cell clones

Tumor tissue was manually minced and cultured in complete AIM-V medium supplemented with liberase (0.1 mg/ml; Roche) and deoxyribonuclease (1.75 μg/ml; Roche) for 30 min. The cells were cultured in complete AIM-V with interleukin-2 (IL-2) (6000 U/ml; Peprotech) and amphotericin B (2.5 μg/ml; Gibco) for 4 weeks, and cultured in complete AIM-V supplemented with IL-2 (6000 U/ml), anti-CD3 (30 ng/ml; OKT3, BioLegend 317302), and amphotericin B (2.5 μg/ml) with irradiated (100 Gy) healthy donor–derived PBMCs for an additional 2 weeks. The expanded cells were cryopreserved and used as bulk TILs. Bulk TILs were cultured with T2 cells pulsed with 1 μM synthetic peptide (neoantigen), and CD8 and 4-1BB (CD137)–double-positive cell populations were isolated using FACSAria II (BD) and sorted into U-bottom 96-well plates. Each single cell was expanded in complete AIM-V medium supplemented with IL-2 (3000 U/ml), phytohemagglutinin (5 μg/ml; WAKO) and irradiated healthy donor PBMCs. The expanded cells were cryopreserved and used as neoantigen-reactive CD8^+^ T cell clones.

### TCR sequencing of T cell clones

TCR clonotypes were determined as previously described (*22*). Briefly, TCRα and TCRβ sequences were isolated from a KRV9-reactive T cell clones or KVI10-reactive T cell clones by 5′-rapid amplification of cDNA ends (RACE) PCR using a SMARTer RACE 5′/3′ kit (Takara Bio) according to the manufacturer’s instructions. The PCR products were cloned into a pMX retroviral vector using the NEBuilder HiFi DNA Assembly Master Mix (NEB). TCRα and TCRβ sequences were sequenced at the Biomedical Research, Education and Instrumentation Center of Sapporo Medical University. The TCR clonotypes were determined using the IMGT database (www.imgt.org).

### Cytokine production by TILs and T cell clones

Intracellular IFN-γ production by bulk TILs was assessed using Fixation/Permeabilization Solution Kit with BD GolgiPlug (BD), according to the manufacturer’s instructions. Briefly, T2 cells were pulsed with 1 μM of the indicated synthetic peptides for 2 hours at 25°C, and TILs were cultured with the T2 cells for 4 hours at 37°C in the presence of human FcR blocking reagent (Clear Back, MBL MTG-001), GolgiPlug, monensin, and anti-CD107a phycoerythrin (PE) (H4A3, BioLegend 328608). TILs were stained with anti-CD3 PE-Cy7 (SK7, BD 341091) and anti-CD8 allophycocyanin (APC) (HIT8a, BioLegend 300911), followed by permeabilization and staining with anti–IFN-γ FITC (4S.B3, BioLegend 502506), and analyzed using a FACSCanto II (BD) with FACSDiva (BD).

IFN-γ production of CD8^+^ T cell clones was assessed by enzyme-linked immunospot (ELISPOT) assay. T2 cells were incubated with 3 nM of the indicated synthetic peptides. T cell clones were mixed with T2 cells at a fixed ratio (1:1) or at indicated ratio and cultured on a human IFN-γ ELISPOT plate (BD) for 24 hours at 37°C. The contents of the plate were reacted with a biotinylated anti-human IFN-γ for 2 hours at 25°C, followed by ELISPOT Streptavidin–horseradish peroxidase for 1 hour at 25°C. Positive spots were visualized using the ELISPOT AEC Substrate Set (BD).

### Lactate dehydrogenase cytotoxicity assay

T2 cells preincubated with or without 1 μM synthetic peptides (KVI10 or KII10) for 2 hours at room temperature served as target cells. Target cells (5.0 × 10^3^) were cultured with CD8^+^ T cell clones (clones 77, 21, and 63) at the indicated effector/target ratios for 8 hours at 37°C. The amount of lactate dehydrogenase (LDH) released from lysed target cells was measured using the LDH Cytotoxicity Detection Kit (Takara Bio) according to the manufacturer’s instructions. LDH release (%) was calculated as previously reported (*44*).

### Cytokine production by neoantigen-reactive CD4^+^ T cells

Patient PBMCs were stimulated with 1 μM each of a mixture of candidate neoantigen peptides on days 0 and 7, and IL-2 (50 U/ml) was added on day 1. On day 14, intracellular production of IFN-γ and TNFα was assessed using Fixation/Permeabilization Solution Kit with BD GolgiPlug (BD), as described above. Briefly, stimulated PBMCs were pulsed with 1 μM of the indicated synthetic peptides in the presence of GolgiPlug and Monensin and cultured for 4 hours at 37°C. PBMCs were then stained with human FcR blocking reagent (Clear Back, MBL MTG-001), anti-CD3 APC-H7 (SK7, BD 641397) and anti-CD4 PE-Cy7 (OKT4, BioLegend 317414), followed by permeabilization and staining with anti–IFN-γ FITC (4S.B3, BioLegend 502506) and anti-TNFα PE (Mab11, BioLegend 502908), and analyzed using FACSCanto II (BD) with FACSDiva (BD).

HLA-II restriction of the T cell responses was validated using a DuoSet enzyme-linked immunosorbent assay (ELISA) kit (R&D Systems). Patient-derived LCL was incubated with 3 μM of the indicated peptides for 2 hours at 25°C. LCL was further incubated with indicated HLA-I or HLA-II antibodies (100 μg/ml) for 1 hour at 37°C. Stimulated PBMCs were then mixed with the LCL at a 1:1 ratio and cultured on a 48-well plate for 24 hours at 37°C. IFN-γ or TNFα in supernatants was quantified using ELISA, according to the manufacturer’s protocol.

### Synthetic peptides

The following synthetic peptides were synthesized with >80% purity (Genscript): KLGEIITTI, KII9; KLNPQRFEV, KRV9; KLNPQQFEV, KQV9; CKIEELYASL, CL10; KLWDIVNVNI, KVI10; KLWDIINVNI, KII10; KGEIAASIVTHMRPY, KVY15; APERILDRCSSILLHS, AS16; TECVRLVTRYIYN, TCN13; TAGGVMTVLINRNTTIPT, TNT18; IPCPRIDTQQLDQQIK, IQK16; VELKIVADQLCAK, VK13; KGEIAASIATHMRPY, KAY15; QPVSSLRF, QSF8; KYLSVQSQL, KSL9; KYIEIYVQKI, KI10; HPQLPYCVVQF, HPF11; SLYNTVATL, HIV.

### Neoantigen vaccination

BALB/c mice were purchased from Hokudo (Sapporo, Japan) and maintained at the animal facility of Sapporo Medical University. On days −10 and −3, mice were subcutaneously injected with 100 μg of poly (I:C) (InvivoGen) containing 50 μg each of synthetic neoantigen peptides or 100 μg of poly (I:C) alone. On day 0, 5.0 × 10^6^ CT26 cells were injected subcutaneously in a mixture of saline and Matrigel (Corning). For ICB monotherapy or combination therapy, 10 mg/kg of anti–PD-1 (29F.1A12, Bio X Cell BE0273) was injected intraperitoneally on days 7 and 14. Tumors were measured every 2 to 3 days, and their sizes were calculated as (*xy*^2^)/2, where *x* and *y* represent the major and minor tumor axes, respectively. The endpoint was set as the major axis of 20 mm. Following antibodies were used in the intracellular cytokine staining of spleen cells and flow cytometry: Fc Block (anti-CD16/CD32, BD 553142), anti-CD107a FITC (1D4B, BioLegend 121606), anti-CD3 PE (17A2, BioLegend 100206), anti-CD8 APC (53-6.7, BioLegend100712), and anti–IFN-γ PE-Cy7 (XMG1.2, BioLegend 505826). For blocking assay using CD8 or CD4 antibodies, syngeneic BALB/c mice were injected subcutaneously with 50 μg of KSL9 peptide, intraperitoneally with PD-1 antibody (10 mg/kg) or 200 μg of anti-CD8 (2.43, Bio X Cell BE0061) or anti-CD4 (GK1.5, Bio X Cell BE0003-1) or isotype-matched immunoglobulin G antibody (LTF-2, Bio X Cell BE0090). Mice were injected subcutaneously with 5.0 × 10^6^ CT26 cells on day 0, and their sizes were monitored. All procedures were performed in accordance with the institutional animal care guidelines. This study was approved by the Animal Study Committee of Sapporo Medical University (22-053).

### Tandem IVTT for immunogenic neoantigen screening

DNA vectors were designed to encode all HLA-A*02:01-bound neoantigen sequences detected by in silico prediction of CRC135 mutation data with %rank NetMHC scores below 0.5. Up to 10 candidate neoantigen units were inserted in tandem per single pMA-RQ or pOA-RQ vector (GeneArt, Thermo Fisher Scientific). Each neoantigen unit contained an upstream T7 promoter, ribosome binding site, start codon and FLAG sequence, and a downstream stop codon and 3′UTR stem loop (fig. S2A). Each neoantigen unit was flanked by unique restriction enzyme recognition sites as shown in fig. S2B. For the first round of screening, bulk peptide pools were directly transcribed and translated from 1 μg of each vector using the PURExpress In Vitro Protein Synthesis Kit (NEB) in the presence of murine ribonuclease (RNase) inhibitor (NEB) and enterokinase (NEB). Enterokinase was used to separate each peptide from the upstream FLAG sequence after translation. For the second round of screening, each neoantigen sequence was digested from 2 μg of a selected vector by coincubation with the appropriate restriction enzyme pairs for 2.5 hours at 37°C, separated by electrophoresis in 4% NuSieve GTG agarose gel (Lonza), and extracted and purified using the FastGene Gel/PCR Extraction Kit (Nippon Genetics). Individual peptides were then separately translated using the PURExpress In Vitro Protein Synthesis Kit (NEB) in the presence of murine RNase inhibitor (NEB) and enterokinase (NEB). For both screens, T cell responses were evaluated against T2 cells pulsed with the IVTT products.

### Statistics

Data analysis was performed using the GraphPad Prism software (v8). Data are represented as the means ± SEM.
