# Supplementary material for: Identification of immunogenic HLA class I and II neoantigens using surrogate immunopeptidomes
Source: Sci Adv. 2024 Sep 18;10(38):eado6491. doi: 10.1126/sciadv.ado6491 (PMC11409964; doi:10.1126/sciadv.ado6491)
Supplement: Supplementary file 1 — Figs. S1 to S7 Legends for tables S1 to S5 [file sciadv.ado6491_sm.pdf]

Science Advances
AAAS

Supplementary Materials for

Identification of immunogenic HLA class I and II neoantigens using surrogate immunopeptidomes

Serina Tokita *et al.*

Corresponding author: Takayuki Kanaseki, kanaseki@sapmed.ac.jp

Sci. Adv. **10**, eado6491 (2024)
DOI: 10.1126/sciadv.ado6491

The PDF file includes:

Figs. S1 to S7
Legends for tables S1 to S5

Other Supplementary Material for this manuscript includes the following:

Tables S1 to S5

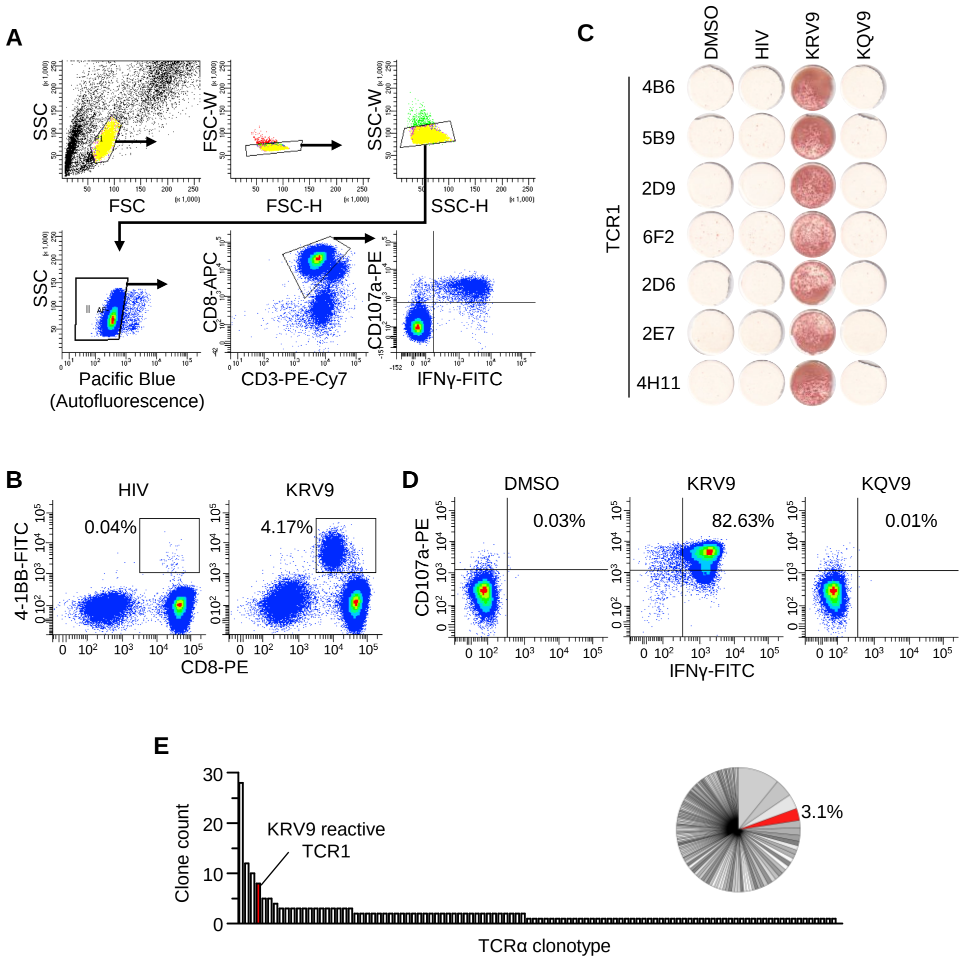

Fig. S1 | Neoantigen (KRV9)-reactive $CD8^+$ T cell clones.

A, Gating strategies to assess the neoantigen reactivity of CRC135 bulk TILs using flow cytometry. **B**, Flow cytometry of CRC135 bulk TILs. Numbers in the rectangles indicate the proportion of 4-1BB$^+$ cells in $CD3^+$ $CD8^+$ cells. TILs were analyzed in the presence of T2 cells pulsed with the indicated peptides. Data are representative of two independent experiments. **C**, IFNγ ELISPOT assay of KRV9-reactive $CD8^+$ T cell clones (4B6, 5B9, 2D9, 6F2, 2D6, 2E7, and 4H11) with the same TCRαβ clonotype (TCR1) sorted from CRC135 bulk TILs in response to T2 cells pulsed with 100 nM of the indicated peptides. Data are representative of four independent experiments. **D**, Frequency of IFNγ$^+$ CD107a$^+$ cells recognizing the indicating peptide in the 4H11 clone. Data are representative of two independent experiments. **E**, Distribution (bar chart) and frequency (pie chart) of TCRα clonotypes in CRC135 bulk TILs (136 clonotypes in total). TCRα clonotype of the KRV9-reactive $CD8^+$ T-cell clones (TCR1) is shown in red.

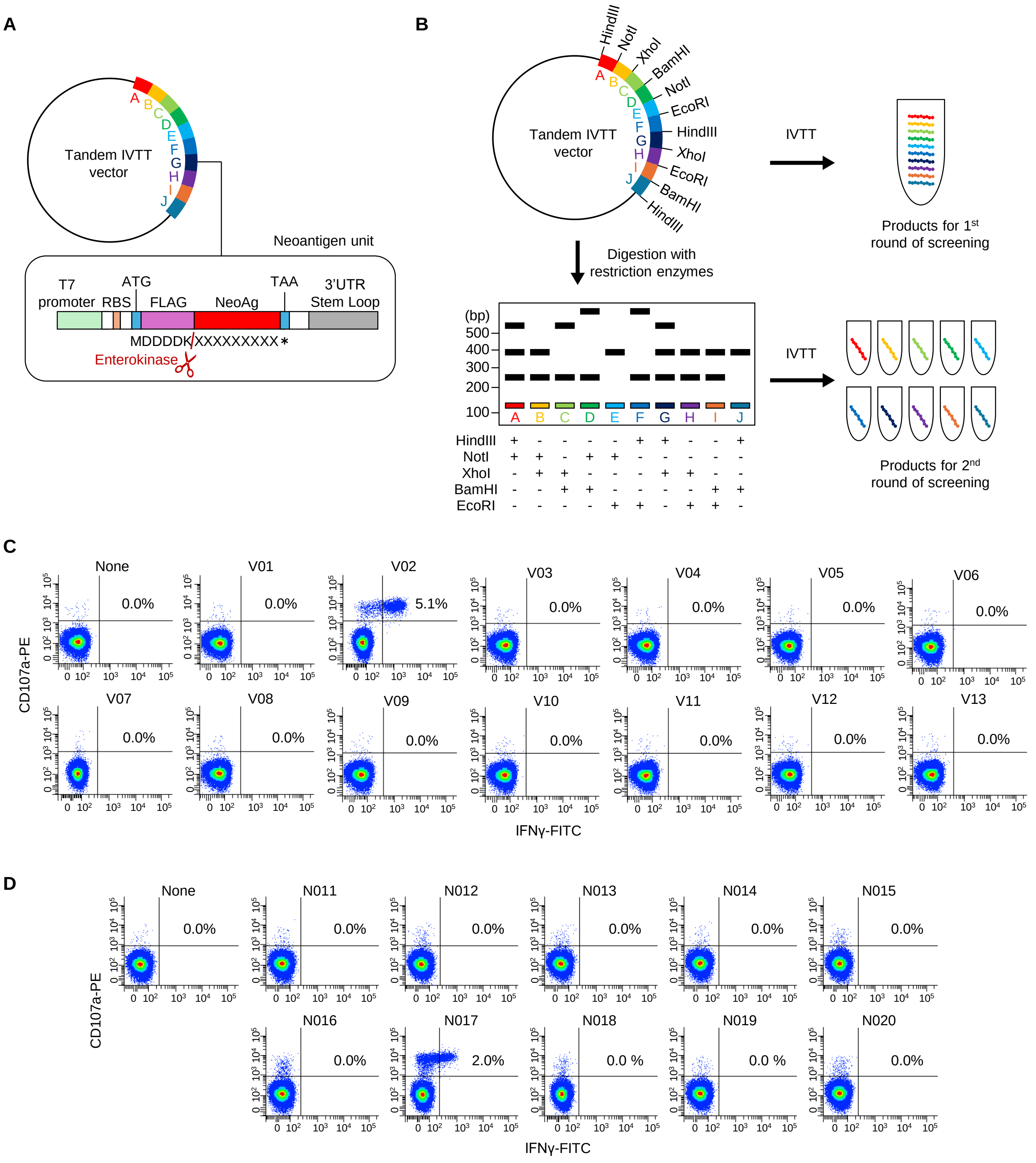
A
Tandem IVTT vector
A B C D E F G H I J
Neoantigen unit
T7 promoter
RBS
ATG
FLAG
NeoAg
TAA
3'UTR Stem Loop
MDDDDK XXXXXXXXX *
Enterokinase
B
HindIII
NotI
XhoI
BamHI
NotI
EcoRI
HindIII
XhoI
EcoRI
BamHI
HindIII
Tandem IVTT vector
IVTT
Products for 1st round of screening
Digestion with restriction enzymes
(bp)
500
400
300
200
100
A B C D E F G H I J
HindIII + - - - - + + - - +
NotI + + - + + - - - - -
XhoI - + + - - - + + - -
BamHI - - + + - - - - + +
EcoRI - - - - + + - + + -
IVTT
Products for 2nd round of screening
C
None 0.0%
V01 0.0%
V02 5.1%
V03 0.0%
V04 0.0%
V05 0.0%
V06 0.0%
V07 0.0%
V08 0.0%
V09 0.0%
V10 0.0%
V11 0.0%
V12 0.0%
V13 0.0%
CD107a-PE
IFNγ-FITC
D
None 0.0%
N011 0.0%
N012 0.0%
N013 0.0%
N014 0.0%
N015 0.0%
N016 0.0%
N017 2.0%
N018 0.0 %
N019 0.0 %
N020 0.0%
CD107a-PE
IFNγ-FITC

Fig. S2 | Neoantigen screening using tandem IVTT.

A, Blueprint of a tandem IVTT vector. One vector contained up to 10 neoantigen units (shown as A to J), each unit consisting of a neoantigen coding sequence (shown as NeoAg) with an upstream T7 promoter, ribosome binding site (RBS), start codon and FLAG sequence, and a downstream stop codon and 3'UTR stem loop. **B**, Preparation of IVTT products. For the 1st round screening, a mixed pool of neoantigen peptides was generated from each vector by IVTT. For the 2nd round screening, each neoantigen sequence was digested with the indicated restriction enzymes, separated and extracted by electrophoresis, and separately transcribed and translated by IVTT. **C**, Flow cytometry of $CD8^+$ bulk CRC135 TIL products for the 1st round of screening. Intracellular IFNγ production and CD107a surface expression were measured in response to T2 cells pulsed with the IVTT products of the tandem IVTT vectors (V01 to V13). **D**, Flow cytometry of $CD8^+$ bulk CRC135 TIL products for the 2nd round of screening. Intracellular IFNγ production and CD107a surface expression were measured in response to T2 cells pulsed with the IVTT products of vector V02 consisting of 10 unique neoantigens (N011 to N020). Numbers indicate frequency (%) of $IFN\gamma^+$ $CD107a^+$ cells in $CD3^+$ $CD8^+$ cells.

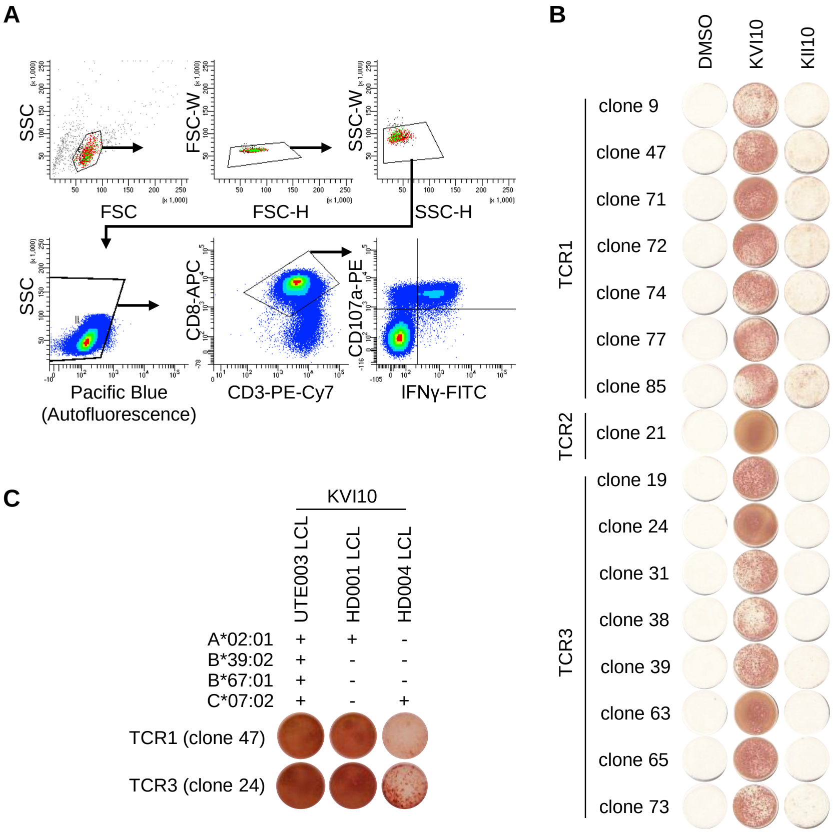

Fig. S3 | Neoantigen (KVI10)-reactive CD8⁺ T cell clones.

A, Gating strategies to assess the neoantigen reactivity of UTE003 bulk TILs by flow cytometry. **B**, IFNγ ELISPOT assay of KVI10-reactive CD8$^+$ T cell clones with different TCRαβ clonotypes derived from UTE003 TILs in the presence of 1 μM of the indicated peptides. Data are representative of two independent experiments. **C**, IFNγ ELISPOT assay of clones 47 and 24 against a panel of LCLs pulsed with 1 μM KVI10 peptide, indicating the HLA-A*02:01 restriction of T cell responses to KVI10. Data are representative of two independent experiments.

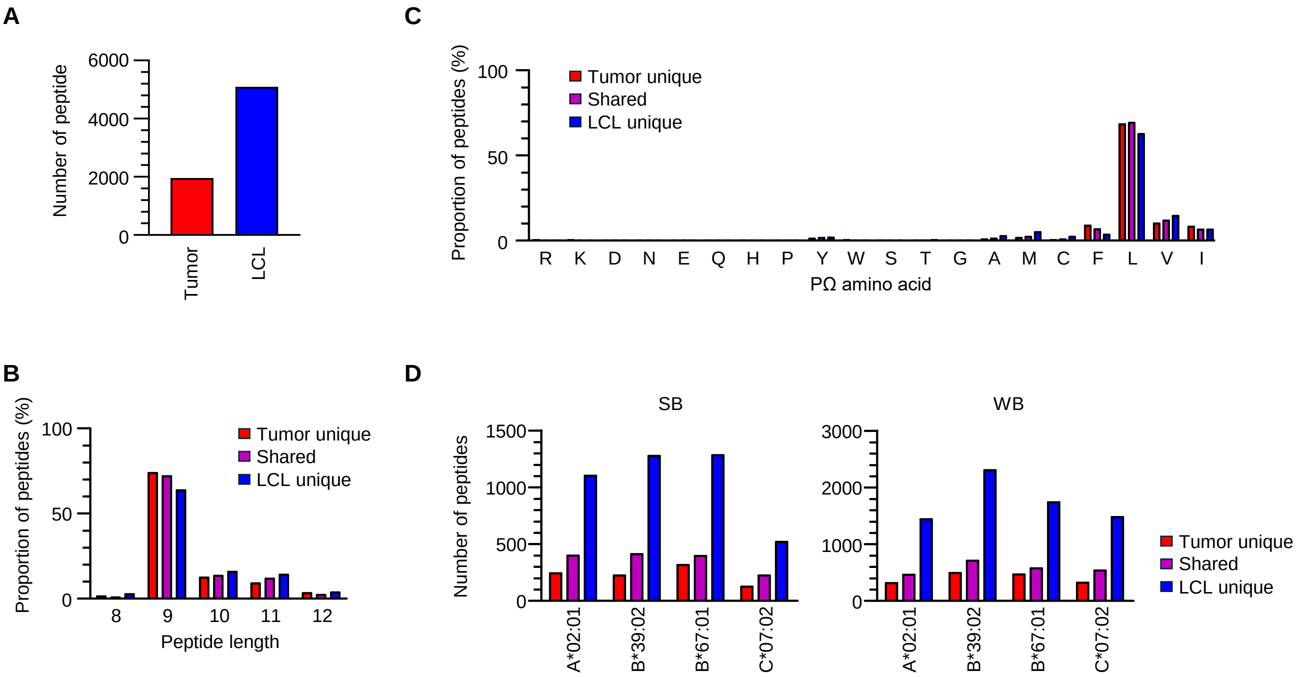

Fig. S4 | Quality comparison between tumor and LCL immunopeptidomes.

A, Total number of non-redundant peptides in the UTE003 tumor and LCL HLA-I immunopeptidomes. **B**, Length distribution of peptides unique to or shared between the UTE003 tumor and LCL immunopeptidomes. **C**, Proportion of C-terminal amino acid composition of peptides unique to or shared between the UTE003 tumor and LCL immunopeptidomes. **D**, Number of peptides unique to or shared between the UTE003 tumor and LCL immunopeptidomes after HLA binding motif deconvolution. Bar graphs show the number of peptides classified to one or more UTE003 HLA-I genotypes according to NetMHCpan-4.1 %rank scores (SB, strong binders with %rank scores < 0.5; WB, weak binders with %rank scores < 2.0).

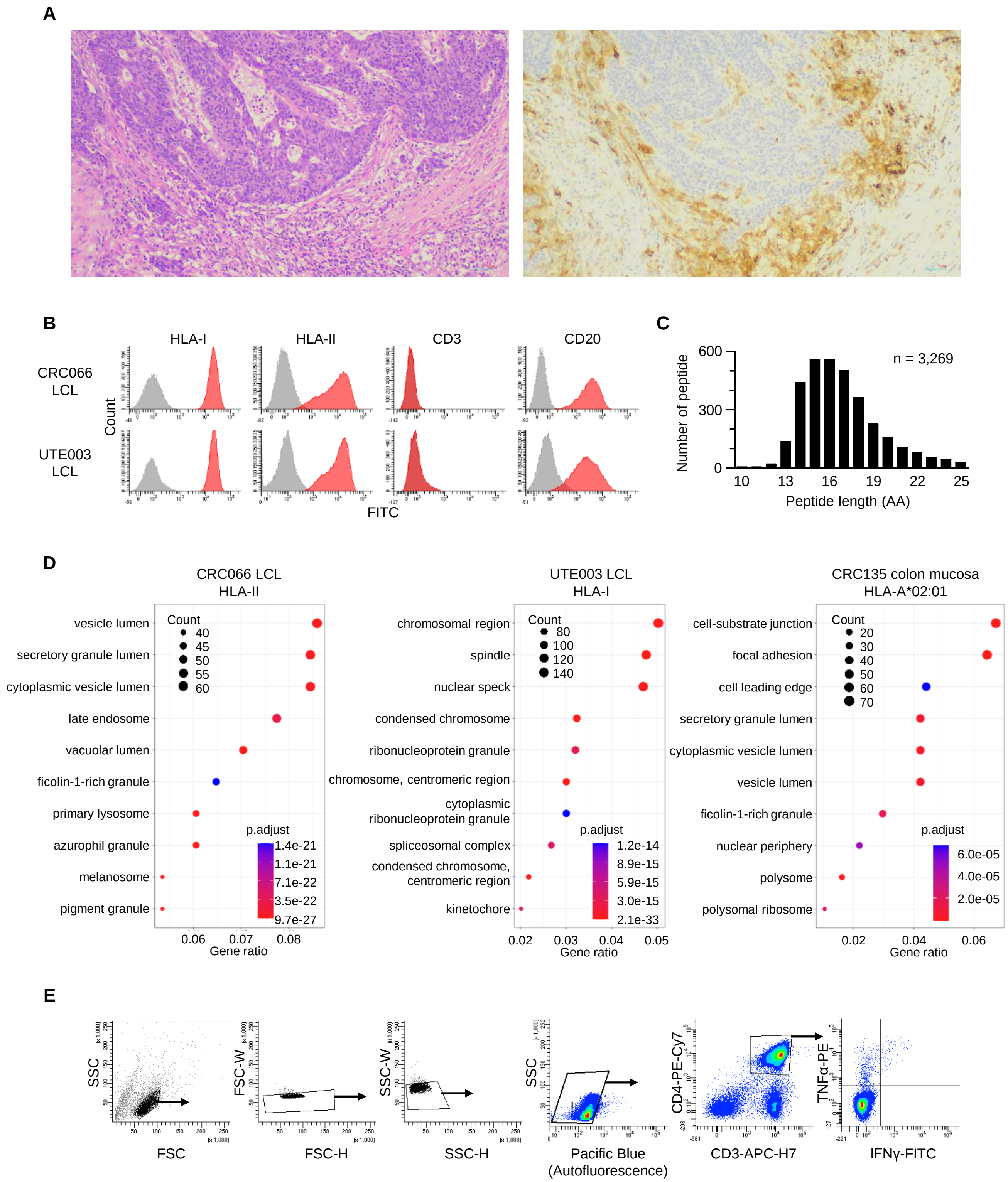
A
B
HLA-I
HLA-II
CD3
CD20
CRC066 LCL
UTE003 LCL
Count
FITC
C
Number of peptide
n = 3,269
600
300
0
10
13
16
19
22
25
Peptide length (AA)
D
CRC066 LCL HLA-II
vesicle lumen
secretory granule lumen
cytoplasmic vesicle lumen
late endosome
vacuolar lumen
ficolin-1-rich granule
primary lysosome
azurophil granule
melanosome
pigment granule
Count
40
45
50
55
60
p.adjust
1.4e-21
1.1e-21
7.1e-22
3.5e-22
9.7e-27
0.06
0.07
0.08
Gene ratio
UTE003 LCL HLA-I
chromosomal region
spindle
nuclear speck
condensed chromosome
ribonucleoprotein granule
chromosome, centromeric region
cytoplasmic ribonucleoprotein granule
spliceosomal complex
condensed chromosome, centromeric region
kinetochore
Count
80
100
120
140
p.adjust
1.2e-14
8.9e-15
5.9e-15
3.0e-15
2.1e-33
0.02
0.03
0.04
0.05
Gene ratio
CRC135 colon mucosa HLA-A*02:01
cell-substrate junction
focal adhesion
cell leading edge
secretory granule lumen
cytoplasmic vesicle lumen
vesicle lumen
ficolin-1-rich granule
nuclear periphery
polysome
polysomal ribosome
Count
20
30
40
50
60
70
p.adjust
6.0e-05
4.0e-05
2.0e-05
0.02
0.04
0.06
Gene ratio
E
SSC
FSC
FSC-W
FSC-H
SSC-W
SSC-H
SSC
Pacific Blue (Autofluorescence)
CD4-PE-Cy7
CD3-APC-H7
TNFα-PE
IFNγ-FITC

Fig. S5 | HLA-II immunopeptidome of LCLs.

A, Immunohistochemistry showing HLA-II negative tumor cells surrounded by HLA-II positive stromal cells. Formalin-fixed paraffin-embedded tissues of CRC066 tumor were mounted and stained with hematoxylin and eosin (left) or anti-pan HLA-II (IVA12 (HB-145), ATCC) (right). **B**, Flow cytometry of patient peripheral blood LCLs showing constant HLA-I and HLA-II surface expression. **C**, Length distribution of HLA-II ligands identified in CRC066 LCLs. **D**, Dot plots showing the Gene Ontology (GO) cellular components enriched in the source genes of the indicated immunopeptidomes, suggesting the enrichment in extracellular or membrane proteins in HLA-II ligands. Size and color of the dots represent the gene count and significance level, respectively. Analysis was conducted using the R package (clusterProfiler). **E**, Gating strategies to assess the neoantigen reactivity of CRC066 PBMCs by flow cytometry.

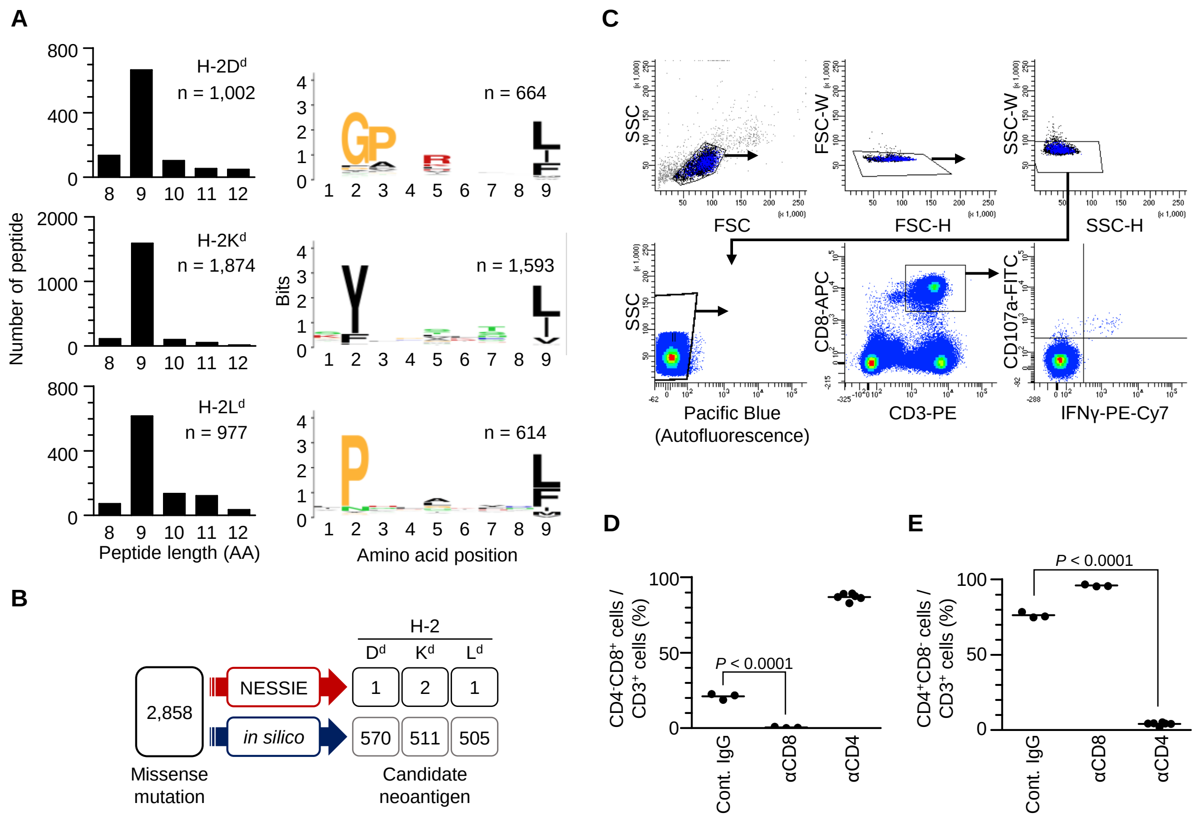

Fig. S6 | Identification of an immunogenic neoantigen in a mouse tumor model.

A, Length distribution (left) and sequence logos of 9-mer peptides (right) of H-2D^d, K^d, and L^d wild-type immunopeptidomes of BALB/c spleen cells used for NESSIE identification of neoantigens in a CT26 tumor model. Patterns of amino acid sequence preservation at positions 2 and 9 indicate discrimination and successful elution of peptides bound to each MHC I allele type. **B**, CT26 neoantigens detected by NESSIE or in silico prediction (%rank score of NetMHCpan4.1 < 2.0). Only peptides with 8-12 mer and gene expression (TPM > 1) were selected for both NESSIE and in silico prediction. Number of non-redundant candidate neoantigens is shown. **C**, Gating strategies to assess the neoantigen reactivity of spleen cells using flow cytometry. **D and E**, Flow cytometry of spleen cells showing the frequency of CD4$^-$ CD8$^+$ (**D**) or CD4$^+$ CD8$^-$ (**E**) cells in CD3$^+$ after the administration of the blocking antibodies. p-values were calculated using a two-tailed unpaired t-test.

A

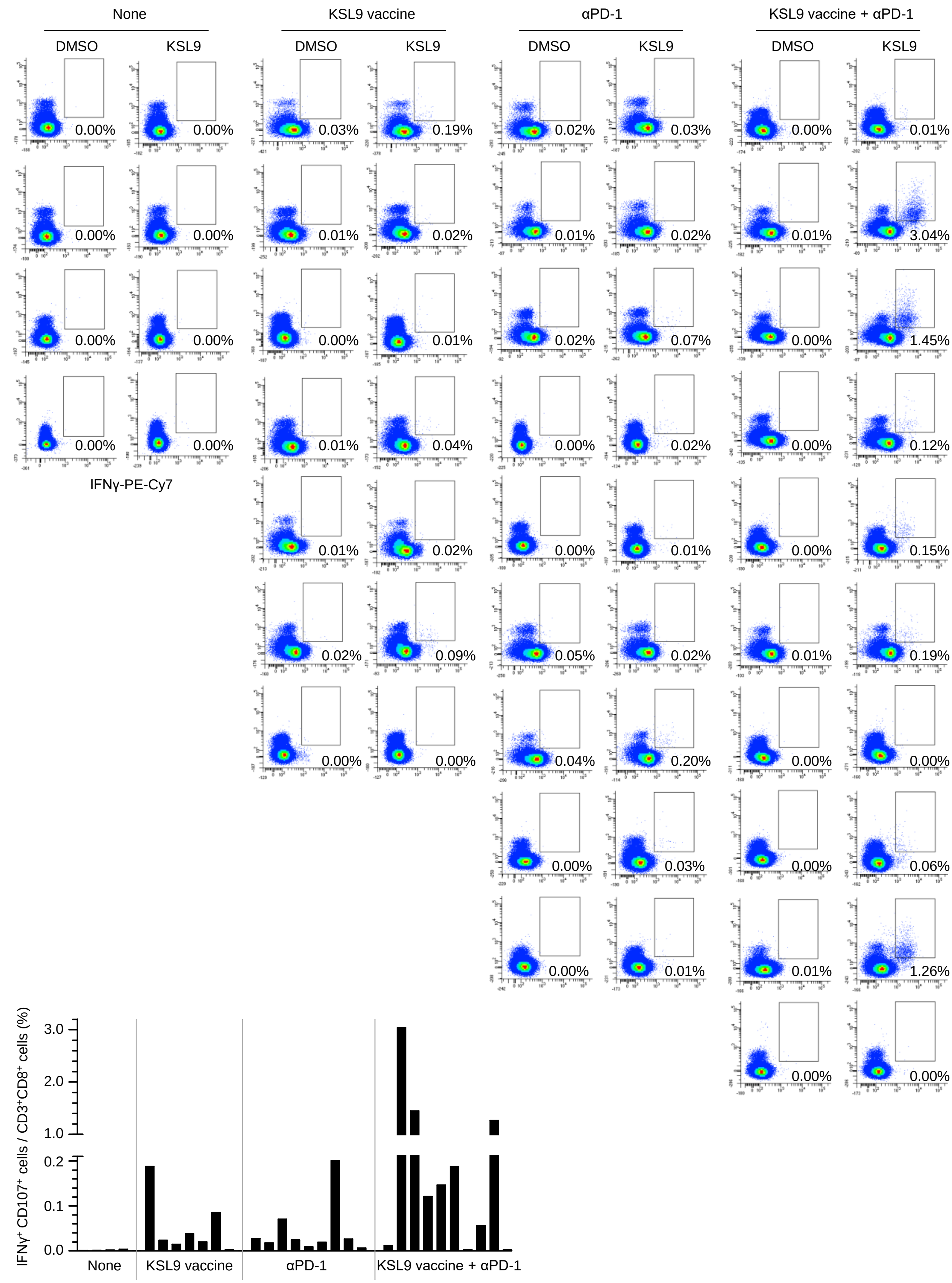
None
KSL9 vaccine
αPD-1
KSL9 vaccine + αPD-1
DMSO
KSL9
CD107a-FITC
IFNγ-PE-Cy7
0.00% 0.00% 0.03% 0.19% 0.02% 0.03% 0.00% 0.01%
0.00% 0.00% 0.01% 0.02% 0.01% 0.02% 0.01% 3.04%
0.00% 0.00% 0.00% 0.01% 0.02% 0.07% 0.00% 1.45%
0.00% 0.00% 0.01% 0.04% 0.00% 0.02% 0.00% 0.12%
0.01% 0.02% 0.00% 0.01% 0.00% 0.15%
0.02% 0.09% 0.05% 0.02% 0.01% 0.19%
0.00% 0.00% 0.04% 0.20% 0.00% 0.00%
0.00% 0.03% 0.00% 0.06%
0.00% 0.01% 0.01% 1.26%
0.00% 0.00%
B
IFNγ+ CD107+ cells / CD3+CD8+ cells (%)
3.0
2.0
1.0
0.2
0.1
0.0
None
KSL9 vaccine
αPD-1
KSL9 vaccine + αPD-1

Fig. S7 | Systemic induction of neoantigen-reactive T cells by combined vaccination and ICB.

A, Flow cytometry of $CD8^+$ spleen cells after the administration of KSL9 vaccine alone (n = 7), PD-1 antibody alone (n = 9), or both (n = 10) with a control group (n = 4). Intracellular IFNγ production and CD107a surface expression in response to KSL9 peptides were measured as described in the Methods section. **B**, Bar graph representing the frequency of KSL9-reactive $CD8^+$ T cells shown in Fig. S7A.

Table S1 | HLA-A*24:02 peptidomes of CRC tumor tissues

Table S2 | CRC135: Neoantigens detected by NESSIE, HLA-A*02:01 peptidome of colon mucosa tissue used for NESSIE search, neoantigen-reactive TCRs sequences, and list of in silico predicted neoantigens with %rank score below 0.5

Table S3 | UTE003: Neoantigens detected by NESSIE, HLA-I peptidome of patient LCL used for NESSIE search, and neoantigen-reactive TCRs sequences

Table S4 | CRC066: Neoantigens detected by NESSIE, HLA-II peptidome of patient LCL used for NESSIE search

Table S5 | CT26: Neoantigens detected by NESSIE, MHC-I peptidomes of BALB/c spleen cells used for NESSIE search
